# Targeting Copper Homeostasis Improves Functioning of *vps13*Δ Yeast Mutant Cells, a Model of *VPS13*-Related Diseases

**DOI:** 10.3390/ijms22052248

**Published:** 2021-02-24

**Authors:** Piotr Soczewka, Déborah Tribouillard-Tanvier, Jean-Paul di Rago, Teresa Zoladek, Joanna Kaminska

**Affiliations:** 1 Institute of Biochemistry and Biophysics, Polish Academy of Sciences, 02-106 Warsaw, Poland; teresa@ibb.waw.pl; 2 IBGC, UMR 5095, CNRS, Université de Bordeaux, F-33000 Bordeaux, France; deborah.tribouillard-tanvier@ibgc.cnrs.fr (D.T.-T.); jp.dirago@ibgc.cnrs.fr (J.-P.d.R.); 3 Institut National de la Santé et de la Recherche Médicale (INSERM), F-33077 Bordeaux, France

**Keywords:** yeast model, neurodegeneration, *VPS13*, *CTR1*, *CCC2*, *FET3*, disulfiram, elesclomol, pyrithione, copper homeostasis

## Abstract

Ion homeostasis is crucial for organism functioning, and its alterations may cause diseases. For example, copper insufficiency and overload are associated with Menkes and Wilson’s diseases, respectively, and iron imbalance is observed in Parkinson’s and Alzheimer’s diseases. To better understand human diseases, *Saccharomyces cerevisiae* yeast are used as a model organism. In our studies, we used the *vps13*Δ yeast strain as a model of rare neurological diseases caused by mutations in *VPS13A*–*D* genes. In this work, we show that overexpression of genes encoding copper transporters, *CTR1*, *CTR3*, and *CCC2*, or the addition of copper salt to the medium, improved functioning of the *vps13*Δ mutant. We show that their mechanism of action, at least partially, depends on increasing iron content in the cells by the copper-dependent iron uptake system. Finally, we present that treatment with copper ionophores, disulfiram, elesclomol, and sodium pyrithione, also resulted in alleviation of the defects observed in *vps13*Δ cells. Our study points at copper and iron homeostasis as a potential therapeutic target for further investigation in higher eukaryotic models of *VPS13*-related diseases.

## 1. Introduction

Copper is a trace element essential for human health. It regulates the activity of many enzymes, called cuproenzymes, in which it serves as a cofactor or allosteric regulator [1]. Cuproenzymes facilitate various reactions that are important for cellular respiration, oxidative stress defence, iron oxidation, melanin synthesis, connective tissue maturation, blood clotting, and lipid metabolism [1,2,3]. Copper is also crucial for nervous system functioning where it is involved in neurotransmission [3]. Lastly, copper binds to the transcription factors and regulates gene expression, mostly involved in copper storage and buffering [1]. To maintain copper physiological levels, copper homeostasis is tightly regulated [4]. Its alterations are often linked with various diseases. Disturbances may result from nutrition-related causes, but also impaired ion homeostasis regulation, which could have a genetic background. For example, mutations in the *ATP7A* gene, encoding a copper transporter responsible for copper transport from enterocytes to blood, lead to copper deficiency and development of Menkes disease characterised by progressive neurodegeneration and connective tissue disturbances [5]. Genetic inactivation of *ATP7B*, an *ATP7A* paralog encoding copper transporter responsible for removing copper excess from the liver to bile, results in copper overload and development of Wilson’s disease, primarily manifesting in liver function impairment [5]. While the disease progresses, copper accumulates in other organs, including the brain, which may cause neurodegeneration [5]. Moreover, copper is also linked with neurodegenerative diseases with protein aggregation. Copper interacts with the amyloid precursor protein, its peptide derivatives and huntingtin, which are involved in Alzheimer’s and Huntington’s diseases, respectively [3]. It is suggested that this interaction mediates protein aggregation and contributes to disease development [3]. Moreover, metals found in synapses, including copper, contribute to α-synuclein aggregation, a hallmark of Parkinson’s disease [6]. Therefore, copper could be a potential factor important for disease progression [6]. On the other hand, copper levels are reduced in the affected brain regions of Parkinson’s disease patients [6]. This shows that function of copper in the brain and its contribution to neurodegenerative diseases is not fully elucidated. To investigate these diseases and to better understand their molecular pathologies, research on laboratory models, including yeast, were carried out.

*Saccharomyces cerevisiae* yeast is a unicellular organism commonly used in research regarding genetics, cell, and molecular biology. This is based on the fact that processes crucial for proper cellular functioning are conserved from yeast to humans. Therefore, it is possible to use yeast for studying human diseases. Yeast models of Parkinson’s, Alzheimer’s, and Huntington’s diseases have been developed [7,8]. In these models, respective protein aggregates are produced and then their impact on cell functioning is investigated. Most importantly, some findings from research on yeast models were confirmed in mammalian cell lines [9,10], which shows yeast usefulness in studies on candidate therapeutic targets and drugs. Moreover, many human genes complement gene deletions in yeast [11], which allows for the use of yeast as a platform for studying the effects of disease-causing mutations. Examples are human *ATP7A* and *ATP7B*, human homologues of the yeast *CCC2* gene [12]. *CCC2* encodes an ATPase, which transports copper to the Golgi apparatus [13,14]. The cDNA of *ATP7A* and *ATP7B* complements *CCC2* deletion [15,16,17,18], and the yeast system is used to study pathogenic mutations in *ATP7A* and *ATP7B* [19,20,21,22]. Lastly, ion regulation is well characterised in yeast [23]; therefore, yeast could be used for studying disturbances of ion homeostasis including copper homeostasis.

In yeast, copper enters the cell mainly via Ctr1 or Ctr3 plasma membrane copper transporters [24,25,26]. However, due to the Ty2 transposon insertion in the *CTR3* promoter region, *CTR3* expression is abolished in S288C-derived laboratory strains including BY4741 [27]. Alternative copper uptake routes using non-specific transporters were also described and are mostly utilised in copper-rich environment, which causes Ctr1 degradation [23,28,29]. Copper is redox active metal, and it can be used in various redox reactions important for cellular physiology. For example, it is involved in iron import. Copper is a cofactor in Fet3 oxidase, which, together with Ftr1 permease, comprises a high-affinity iron uptake system in yeast cells, crucial for growth in iron limited conditions [30,31]. In this system, Fet3 oxidises ferrous ions (Fe^2+^) to ferric ions (Fe^3+^) that are subsequently transported across the plasma membrane [30,32]. Before targeting to the plasma membrane, Fet3 is loaded with copper in the Golgi apparatus [13,14,30]. For that, Ccc2 ATPase is required [13,14]. In addition to the role in iron uptake, copper is also essential for cellular respiration, because it facilitates electron transport in cytochrome c oxidase, the last enzyme of mitochondrial respiratory electron transport chain [33]. Finally, copper is also a cofactor of superoxide dismutase 1 (Sod1), a component of the yeast defence system against reactive oxygen species [34].

In our research, we used a yeast model of rare, neurological diseases associated with mutations in human *VSP13* genes. There are four human homologs (*VPS13A*–*D*) [35], and mutations in these genes result in chorea-acanthocytosis (ChAc) (*VPS13A*) [36,37,38], Cohen’s syndrome (*VPS13B*) [39], early-onset parkinsonism (*VPS13C*) [40] and childhood-onset movement disorder (*VPS13D*) [41,42]. Molecular pathogenesis of these diseases is not understood and no effective treatment is available for the patients. In yeast, one *VPS13* homolog is present. Originally, it was identified as a gene encoding protein involved in vacuolar protein sorting (VPS, origin of the name) [43,44,45]. Other studies showed its involvement in autophagy [46,47], mitochondrial DNA maintenance [47], sporulation [44,48,49], endocytosis [50,51], actin cytoskeleton organization [50] and calcium signalling [51]. Recently, Vps13 proteins have also been shown to localise, both in yeast and mammalian cell lines, at membrane contact sites (MCS) [47,52,53,54]. MCS are zones of close proximity between different organelles or between organelles and the plasma membrane, which enable direct exchange of lipids and small molecules [55]. At MCS, Vps13 protein most likely maintains lipid transport between organelles, since it acts as a lipid transfer protein in in vitro studies and structure of its N-terminal part is predisposed for lipid channelling between membranes [53,56].

Recently, we showed that *vps13* mutant cells are hypersensitive to sodium dodecyl sulphate (SDS), and this phenotype could be used for genetic and chemical suppressors screening [51,57]. Identified suppressors were further analysed to define their mechanisms of action. In this manner, we found that calcineurin downregulation by a *MYO3* gene fragment or FK506 inhibitor overcomes defects observed in the *vps13*Δ strain [51]. We also described flavonoids as a group of compounds which mitigate *vps13*Δ growth defect [57]. Finally, we showed that the *FET4* gene, encoding a low-affinity and low-specificity iron transporter [58,59,60], is a multicopy suppressor of *vps13*Δ, and that iron is important for yeast growth in the presence of SDS [57].

In this work, we present the *CTR3* gene, encoding plasma membrane copper transporter, as one of the suppressors isolated in our multicopy suppressor screen. We also tested the *CTR1* and *CCC2* genes and found that they also acted as suppressors of *vps13*Δ. We propose that their mechanism of action in *vps13*Δ relies on increasing the intracellular iron content via the copper-dependent high-affinity iron uptake system. Finally, we identified three copper ionophores, disulfiram, elesclomol, and sodium pyrithione, as compounds alleviating *vps13*Δ growth defect. These findings show that copper homeostasis and its relevance for iron uptake is an interesting area for further studies in cell lines and higher organism models of *VPS13*-related diseases.

## 2. Results

### 2.1. Overexpression of CTR1 and CTR3 Genes and Copper Salts Addition Suppress the SDS-Hypersensitivity of vps13Δ Cells

**Figure 1:**
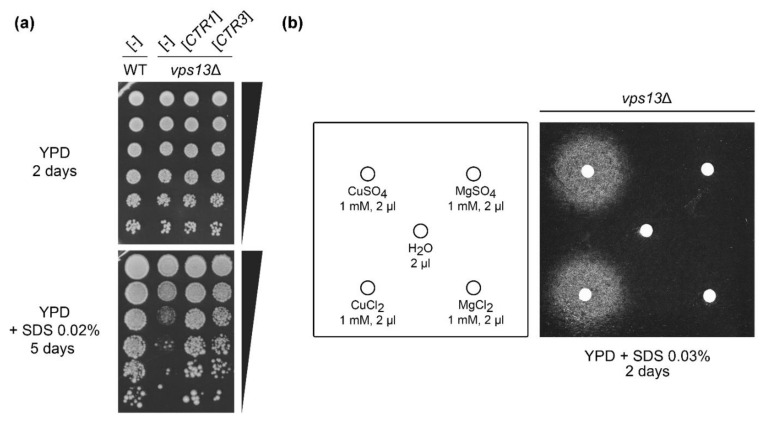
SDS-hypersensitivity phenotype of *vps13*Δ is suppressed by the overexpression of the *CTR1* or *CTR3* genes, encoding copper transporters, and by the addition of copper salts. (**a**) Growth of wild type (WT) and *vps13*Δ strains bearing plasmids with *CTR1* or *CTR3* genes or with empty vector ([-]) was compared by a drop test on plates with YPD and YPD + SDS media. (**b**) The *vps13*Δ strain was spread onto plate with YPD + SDS media and 2 μL of 1 mM solutions of copper sulphate (CuSO_4_), copper chloride (CuCl_2_), magnesium sulphate (MgSO_4_) and magnesium chloride (MgCl_2_) were spotted on the filter papers. Water was used as a negative control.

In our previous research, we discovered an SDS-hypersensitivity growth phenotype of *vps13*Δ mutant and used it to screen for multicopy suppressors [51,57]. One of the identified genes that improved *vps13*Δ growth was *CTR3* (Figure 1a), which encodes a plasma membrane copper transporter [26]. Suppressing plasmid isolated from the pFL-44 genomic bank [61] did not contain Ty2 transposon [27]; therefore, we assumed that *CTR3* expression occurs when *CTR3* was expressed from used vectors. To analyse if suppression is caused by copper uptake or by Ctr3 protein, per se, we checked the *CTR1* gene, encoding the other plasma membrane copper transporter [24]. It also acted as a *vps13*Δ suppressor when overexpressed (Figure 1a). This shows that suppression is related to copper import, not a particular transporter, and further in this study we used *CTR1* as the suppressing gene. To test if copper is important for the suppression, we performed an assay in which *vps13*Δ cells were spread onto YPD + SDS plates (see Materials and Methods), and copper sulphate or copper chloride were applied on the filters placed on the media surface. We observed a growth zones around the filters, which indicate that suppression of *vps13*Δ could also be achieved by treatment with copper salts (Figure 1b). To test if the growth zones were caused by copper cations, not by sulphate or chloride anions, we also checked the impact of magnesium sulphate or magnesium chloride on *vps13*Δ mutant growth. No growth zones were observed around filters with magnesium sulphate and magnesium chloride (Figure 1b). To conclude, these results suggest that increasing copper uptake helps to overcome SDS toxicity in *vps13*Δ cells.

### 2.2. Ctr1–GFP Degradation Is Increased in vps13Δ but vps13Δ Growth Defect Is Not Caused by Copper Deficiency

**Figure 2:**
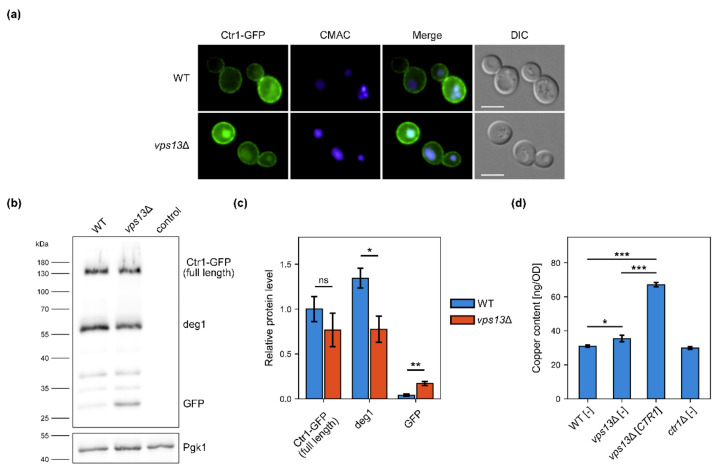
Degradation of Ctr1–GFP and copper level are increased in *vps13*Δ. (**a**) Fluorescence microscopy images of wild type and *vps13*Δ cells with GFP-tagged Ctr1 protein. Vacuoles were stained with CMAC. Scale bar 5 µm. (**b**) Western blot analysis of protein extracts obtained from wild type and *vps13*Δ cells with GFP-tagged Ctr1 protein. Anti-GFP antibody was used for protein detection. Extract from yeast cells without GFP tag was used as a negative control (marked as control). Phosphoglycerate kinase 1 (Pgk1), detected with anti-Pgk1 antibody, was used as a loading control. (**c**) Quantification of western blot results. Relative levels of Ctr1–GFP, deg1, and GFP (in relation to Pgk1) are shown. Mean of the measurements for Ctr1–GFP in wild type cells was set to 1. Results were analysed by two-tailed Student’s *t*-test (*n* = 3, ns *p* > 0.05, * *p* < 0.05, ** *p* < 0.01). Error bars represent SD. (**d**) Copper levels measurement in WT[-], *vps13*Δ[-], *vps13*Δ[*CTR1*] and *ctr1*Δ[-] strains. Yeast were cultivated in YPD + CuSO_4_ media. Copper content was measured by ICP-AES. Obtained results were analysed by ANOVA followed by Tukey’s multiple comparison test (*n* = 3, * *p* < 0.05, *** *p* < 0.001).

Suppression of *vps13*Δ SDS-hypersensitivity by overexpression of genes encoding copper transporters or by copper salts addition may suggest defective copper uptake, which could be caused by impaired action of the Ctr1 in *vps13*Δ cells. We checked if Ctr1 functioning was altered in *vps13*Δ. Firstly, we examined Ctr1 localisation in wild type and *vps13*Δ strains. For that, we tagged the genomic *CTR1* gene with a *GFP*, to produce the Ctr1–GFP protein. In both strains, the GFP signal was detected on the periphery of the cell, suggesting that targeting of Ctr1–GFP to the plasma membrane was not disturbed (Figure 2a). However, we observed cells with a strong GFP signal located in vacuole, stained by the vacuolar marker CMAC (7-amino-4-chloromethylcoumarin), in *vps13*Δ but not in the wild type strain (Figure 2a). This may suggest impaired trafficking, increased turnover or degradation of Ctr1–GFP in *vps13*Δ cells. Next, protein extracts from the wild type and *vps13*Δ cells producing Ctr1–GFP were analysed by the western blot technique using the anti-GFP antibody. Several bands of different molecular weight were detected (Figure 2b), but no band corresponding to protein of predicted molecular weight of Ctr1–GFP (71 kDa) was found. However, in a previous study, a Ctr1–HA protein was shown to migrate in SDS polyacrylamide gel as protein of molecular weight of about 100–105 kDa instead of the predicted 46 kDa [25]. This difference is explained by the heavy glycosylation of Ctr1 [25]. Taking this into account, the band of molecular weight of approximately 130 kDa was likely a full length Ctr1–GFP (Figure 2b). Other bands in the blot were probably products of Ctr1–GFP degradation including the lowest band corresponding to a molecular weight of about 27 kDa, which was most likely free GFP (Figure 2b). Quantification of western blot analysis showed that the level of Ctr1–GFP was lower in *vps13*Δ cells; however, the observed differences were not statistically significant (Figure 2c). Nevertheless, we found significant changes in the levels of degradation products (Figure 2b,c). In *vps13*Δ, the level of one of the degradation product (marked as deg1) and free GFP were, respectively, significantly lower and higher comparing with the wild type strain (Figure 2b,c). The elevated level of the free GFP (Figure 2b,c) corresponded with the strong GFP signal in vacuoles in *vps13*Δ cells (Figure 2a). Because Ctr1–GFP is degraded in the vacuole [29], our results suggest an increased vacuolar degradation of Ctr1–GFP in the *vps13*Δ strain.

**Figure A1:**
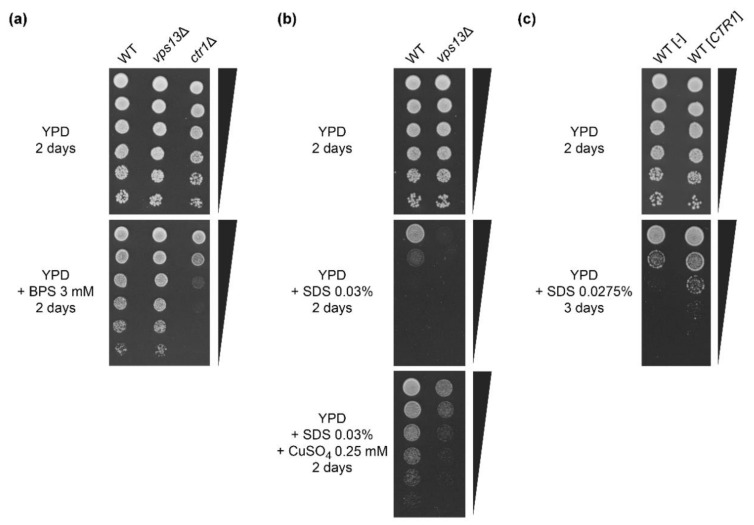
The *vps13*Δ is not sensitive to bathocuproinedisulfonic acid disodium salt (BPS) and copper-based suppression is not specific to *vps13*Δ. (**a**) Growth of wild type, *vps13*Δ and *ctr1*Δ strains was compared by a drop test on plates with YPD and YPD + BPS media. (**b**) Growth of wild type and *vps13*Δ strains was compared by a drop test on plates with YPD, YPD + SDS and YPD + SDS + CuSO_4_ media. (**c**) Impact of *CTR1* overexpression on wild type cells growth was investigated by a drop test on plates with YPD and YPD + SDS media.

Changes observed in the Ctr1–GFP processing in *vps13*Δ cells may result in lowered copper levels. To verify this hypothesis, we measured copper levels in cells of the wild type, *vps13*Δ, *vps13*Δ overexpressing *CTR1* gene and *ctr1*Δ serving as a negative control. For that, we used inductively coupled plasma atomic emission spectroscopy (ICP-AES) technique. We were not able to determine copper levels when yeast were cultivated in YPD media, because the measurement results were below the detection point (not shown). To overcome this, we supplemented YPD media with copper sulphate. When YPD medium was supplemented with copper sulphate, the copper level in *vps13*Δ was slightly elevated in comparison to the wild type strain (Figure 2d). This may suggest a higher demand for copper in *vps13*Δ cells. Overexpression of the *CTR1* gene increased copper level approximately two-fold in *vps13*Δ (Figure 2d), which shows that *CTR1* most likely improves *vps13*Δ growth by increasing intracellular copper. However, the endogenously encoded Ctr1 was not involved in copper import to the yeast cells in tested conditions, because copper levels were comparable in the wild type and *ctr1*Δ cells (Figure 2d). This result was expected, because in copper-rich conditions copper uptake is mediated not by Ctr1 but rather non-specific transporters, since Ctr1 is degraded when copper is abundant [28,29]. Therefore, it is still possible that Ctr1 functioning is impaired in the *vps13*Δ cells, resulting in copper deficiency when copper uptake in yeast cells is mediated by endogenously encoded Ctr1. To address this possibility, we compared the growth of the wild type, *vps13*Δ and *ctr1*Δ strains on the media with bathocuproinedisulfonic acid disodium salt (BPS), a copper chelator, which limits copper bioavailability. Only the *ctr1*Δ strain was sensitive to BPS addition, and the growth of the wild type and *vps13*Δ strains was comparable (Figure A1a). This result shows that Ctr1 functioning is most likely not impaired in *vps13*Δ cells.

The fact that copper levels in *vps13*Δ cells were not lowered but slightly elevated compared to wild type in copper-rich media prompted us to check if *vps13*Δ is still more sensitive to SDS upon copper supplementation. We compared the growth of the wild type and *vps13*Δ cells on YPD + SDS plates with and without copper sulphate addition. Growth of both strains was improved by copper sulphate but *vps13*Δ was still more sensitive to SDS than the wild type (Figure A1b). This result suggests that the *vps13*Δ SDS-hypersensitivity is not caused by copper deficiency. Moreover, copper sulphate improved the growth of the wild type strain, which shows that the suppression is not specific to the *vps13*Δ. We found also that *CTR1* overexpression reduces SDS sensitivity of the wild type strain (Figure A1c).

To conclude, we observed changes in Ctr1–GFP processing indicating its increased degradation in *vps13*Δ cells. We presented that *CTR1* overexpression increases copper content in *vps13*Δ cells. The fact that *vps13*Δ is not sensitive to BPS addition suggest that Ctr1 functioning is not impaired in *vps13*Δ and increased degradation of Ctr1–GFP does not result in copper deficiency. Therefore, the copper-based suppression of *vps13*Δ probably acts by increasing cellular copper level but not by compensation of copper deficiency.

### 2.3. Copper-Dependent High-Affinity Iron Uptake System Is Required for CTR1 Suppression of vps13Δ SDS-Hypersensitivity Phenotype

In our recent study [57], we showed the importance of iron for yeast growth on media with SDS addition. We identified the *FET4* gene, encoding low-affinity iron transporter of plasma membrane, as a multicopy suppressor of *vps13*Δ. The addition of iron salts also improved growth of *vps13*Δ. Moreover, we showed that some yeast mutants defective in iron import are hypersensitive to SDS [57]. Importance of iron for *vps13*Δ growth on SDS media prompted us to check, if increasing copper uptake suppresses *vps13*Δ by increasing iron content in cells. This hypothesis is plausible due to the fact that copper is a cofactor of Fet3 oxidase, a component of the high-affinity iron uptake system. Copper excess caused by *CTR1* overexpression could improve loading of Fet3 with copper, therefore increasing iron import in *vps13*Δ cells. Moreover, it was shown previously that copper supplementation resulted in iron increase in yeast cells [14,62]. This supports the idea of increased copper-dependent iron uptake as a potential suppression mechanism of *vps13*Δ by *CTR1*.

**Figure 3:**
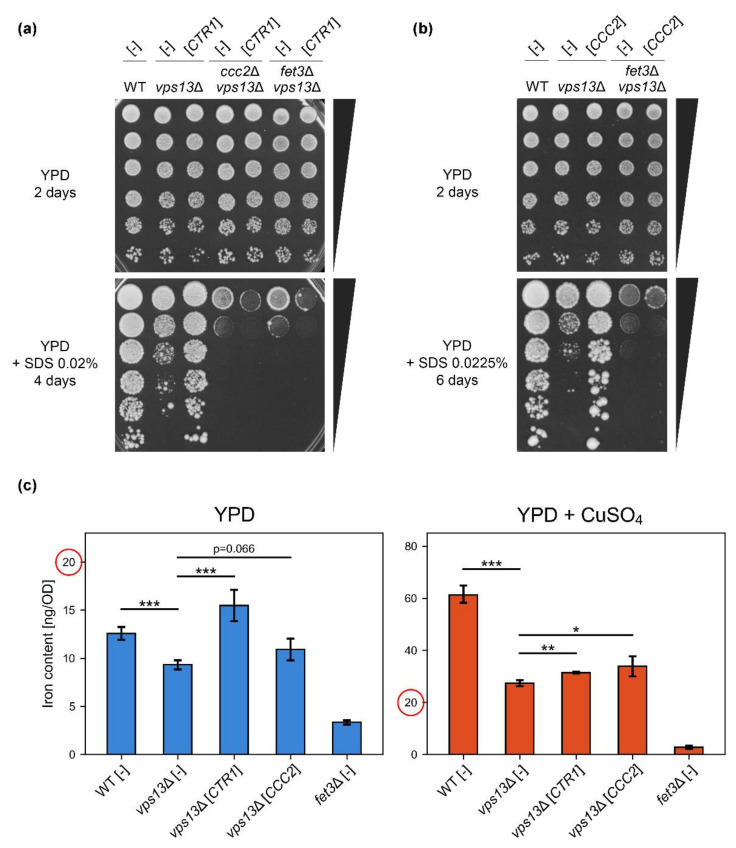
Copper-based suppression of *vps13*Δ SDS-hypersensitivity acts via a high-affinity iron uptake system to increase the iron level in *vps13*Δ. (**a**) Growth of wild type, *vps13*Δ, *ccc2*Δ *vps13*Δ, and *fet3*Δ *vps13*Δ strains bearing plasmid with *CTR1* gene or empty vector was compared by drop test on plates with YPD and YPD + SDS media. (**b**) Growth of wild type, *vps13*Δ and *fet3*Δ *vps13*Δ strains bearing plasmid with the *CCC2* gene or empty vector was compared by a drop test on plates with YPD and YPD + SDS media. (**c**) Iron levels in WT[-], *vps13*Δ[-], *vps13*Δ[*CTR1*], *vps13*Δ[*CTR1*], *vps13*Δ[*CCC2*] and *fet3*Δ[-] (negative control) strains. Iron content was measured in cells of tested strains by ICP-AES. Obtained result were analysed by two-tailed Student’s t-test (*n* = 4, * *p* < 0.05, ** *p* < 0.01, *** *p* < 0.001). Error bars represent SD. Axes are different in panels of c, indicated by red circles on the same position (20) on the scale.

To test our hypothesis, firstly we checked whether the *FET3* and *CCC2* genes, encoding the Fet3 oxidase and Ccc2 copper transporter essential for loading of Fet3 with copper, respectively, were required for *CTR1*-based suppression of *vps13*Δ. We overexpressed *CTR1* in the *ccc2*Δ *vps13*Δ and *fet3*Δ *vps13*Δ strains and tested their growth. Both *ccc2*Δ *vps13*Δ and *fet3*Δ *vps13*Δ were more sensitive to SDS than *vps13*Δ, and *CTR1* overexpression did not improve the growth of any of these strains (Figure 3a). This result shows that the high-affinity iron uptake system is required for *CTR1*-based suppression. Moreover, we observed a slight toxicity caused by *CTR1* overexpression in the double mutants (Figure 3a). Next, we overexpressed the *CCC2* gene in *vps13*Δ cells to check if increased copper import to the Golgi apparatus, potentially increasing Fet3 efficiency, also improves *vps13*Δ growth. Indeed, *CCC2* overexpression suppressed *vps13*Δ but not *fet3*Δ *vps13*Δ (Figure 3b). This suggests that copper ions need to be transported to the Golgi apparatus in order to suppress *vps13*Δ, where most likely they are incorporated into the Fet3 oxidase apoenzyme to increase its activity and, therefore, iron uptake.

To test if overexpression of *CTR1* or *CCC2*, or copper supplementation, increased iron uptake, we performed ICP-AES measurements of iron content in wild type and *vps13*Δ cells bearing empty vector or plasmid expressing *CTR1* or *CCC2* genes that were grown in media with or without copper addition. The *fet3*Δ strain was used as a negative control [30]. The analysis showed that the iron level was lower in *vps13*Δ compared with the wild type, and it significantly increased upon *CTR1* overexpression in *vps13*Δ (Figure 3c, left panel). We observed also a slight increase in iron content when *CCC2* was overexpressed; however, the difference did not cross the statistical significance threshold (*p* = 0.066) (Figure 3c, left panel). Supplementation with copper greatly increased iron content in all tested strains, except *fet3*Δ where the iron level was the lowest in both tested conditions (Figure 3c, compare left and right panels, note that the scales on y-axes are different). Interestingly, the difference in iron levels between the wild type and *vps13*Δ in copper supplemented media was much higher than without copper supplementation: in YPD media, the iron level in *vps13*Δ was 74% of the wild type’s iron level, when in YPD + CuSO_4_ media, it was only 45% (Figure 3c, right panel), suggesting a disturbed copper and iron homeostasis. A slight but statistically significant increase in iron content in *vps13*Δ was observed when *CTR1* or *CCC2* were overexpressed in yeast grown in YPD + CuSO_4_ media (Figure 3c, right panel). Copper supplementation increased iron content also in the wild type cells, which is in line with the fact that copper sulphate improved the growth of wild type strain on YPD + SDS plates (Figure A1b).

To conclude this part, ICP-AES measurements showed a lower iron level in *vps13*Δ and that copper addition, overexpression of *CTR1*, and to some extent *CCC2*, caused its increase. These data, supported by genetic experiments showing requirement of *CCC2* and *FET3* for suppression of *vps13*Δ by *CTR1* (Figure 3a) and by our previous results regarding importance of iron for the growth on YPD + SDS [57], indicate that increasing iron content in *vps13*Δ cells is a likely mechanisms of copper-based suppression of *vps13*Δ.

### 2.4. Cellular Localisation of Fet3–GFP and Ccc2–GFP Proteins Is Not Altered, but Their Levels Are Increased in vps13Δ Cells

**Figure 4:**
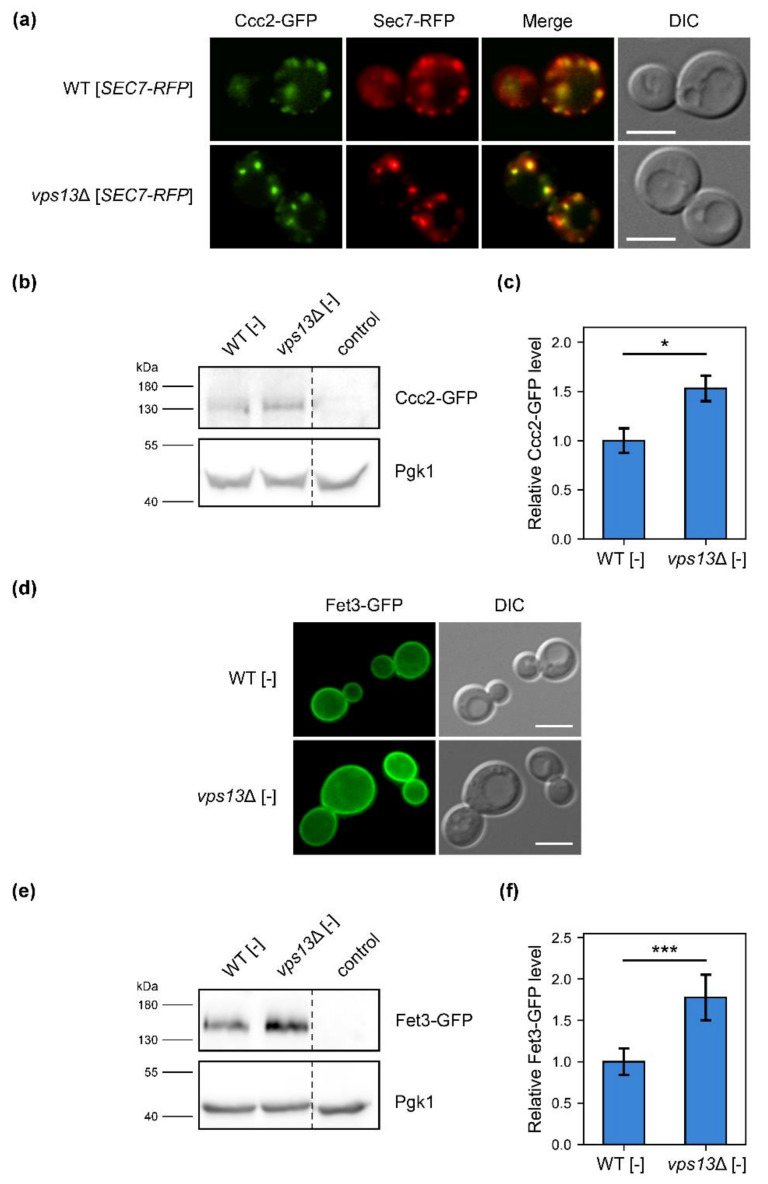
Impact of *VPS13* deletion on Ccc2–GFP and Fet3–GFP localisations and levels. (**a**,**d**) Fluorescence microscopy images of wild type and *vps13*Δ cells with GFP-tagged Ccc2 (**a**) and Fet3 (**d**) proteins. The Sec7–RFP protein was used as a Golgi apparatus marker in Ccc2–GFP visualisation. To maintain the same cultivating conditions, strains with GFP-tagged Fet3 used for microscopic observations were transformed with empty vectors. Scale bar 5 µm. (**b**,**e**) Western blot analysis of protein extracts obtained from yeast with GFP-tagged Ccc2 (**b**) and Fet3 (**e**) proteins. Like in microscopic observations, strains were transformed with empty vector. Anti-GFP antibody was used for protein detection. Pgk1, detected with anti-Pgk1 antibody, was used as a loading control. Extract from yeast cells without GFP tag was used as a negative control (marked as control). (**c**,**f**) Quantification of western blot results. Relative levels of Ccc2–GFP (**c**) and Fet3–GFP (**f**) (in relation to Pgk1) are shown. Results were analysed by two-tailed Student’s t-test (for Ccc2–GFP: *n* = 3 and for Fet3–GFP: *n* = 7; * *p* < 0.05, *** *p* < 0.001). Error bars represent SD.

Requirement for *CCC2* and *FET3* genes for the suppression of *vps13*Δ by *CTR1* overexpression, as well as lower iron level in *vps13*Δ, suggest that the high-affinity iron uptake system may be partially impaired in *vps13*Δ cells. Thus, we checked the functioning of Ccc2 and Fet3 proteins in the *vps13*Δ mutant. For that, we created strains with genomically encoded Ccc2 and Fet3 proteins tagged with GFP fluorescent marker. Firstly, we investigated Ccc2–GFP localisation in wild type and *vps13*Δ cells. To observe if Ccc2–GFP localises to the Golgi apparatus as shown before [14,63], we transformed yeast with plasmid expressing a gene encoding Sec7 tagged with RFP, a marker of the Golgi apparatus late compartments. Colocalisation of GFP and RPF signals was observed in wild type and *vps13*Δ cells (Figure 4a), indicating that Ccc2 was delivered to the Golgi apparatus in both strains. Next, we investigated the level of Ccc2–GFP. Extracts from yeast producing Ccc2–GFP were analysed by the western blot technique using an anti-GFP antibody. We observed a band corresponding to the predicted molecular weight of Ccc2–GFP (137 kDa), and quantification revealed that the Ccc2–GFP level was significantly higher in *vps13*Δ cells compared to the wild type (Figure 4b,c). Increased Ccc2–GFP level in *vps13*Δ cells may indicate increased demand for copper in the Golgi apparatus.

Similarly, we analysed cells with GFP-tagged Fet3 protein. Fluorescence microscopy images showed that—in both wild type and *vps13*Δ cells—Fet3–GFP was targeted to the periphery of cells, suggesting plasma membrane localisation (Figure 4d). In western blot analysis of protein extracts from Fet3–GFP producing strains, we observed the band of a molecular weight of approximately 150 kDa (Figure 4e). In previous studies, the Fet3 protein was shown to migrate in SDS polyacrylamide gel as a protein of molecular weight of about 120 kDa [13,14], instead of the predicted 72 kDa. This difference, alike for Ctr1, is explained by the glycosylation of Fet3 [13,14]. Therefore, the observed band in our western blot is most likely Fet3–GFP (Figure 4e). Quantification of the western blot results showed that Fet3–GFP level is also increased in *vps13*Δ (Figure 4f), comparable to Ccc2–GFP.

To summarise, our results show that the localisation of Ccc2–GFP and Fet3–GFP was properly maintained in *vps13*Δ cells, since both proteins were found at sites of their action. However, the levels of both proteins were increased in *vps13*Δ. This may suggest greater demand for iron in *vps13*Δ.

### 2.5. Copper Ionophores Are Suppressors of vps13Δ

Recently, we performed a screen of the Prestwick Chemical Library to find substances that suppress the *vps13*Δ SDS-hypersensitivity phenotype [57]. Based on the screen results and literature, we selected seven compounds that were active in *vps13*Δ cells. Two of them, luteolin and tolcapone, were described in our previous report [57]. The other three identified compounds were copper ionophores, one of which belongs to the library.

**Figure 5:**
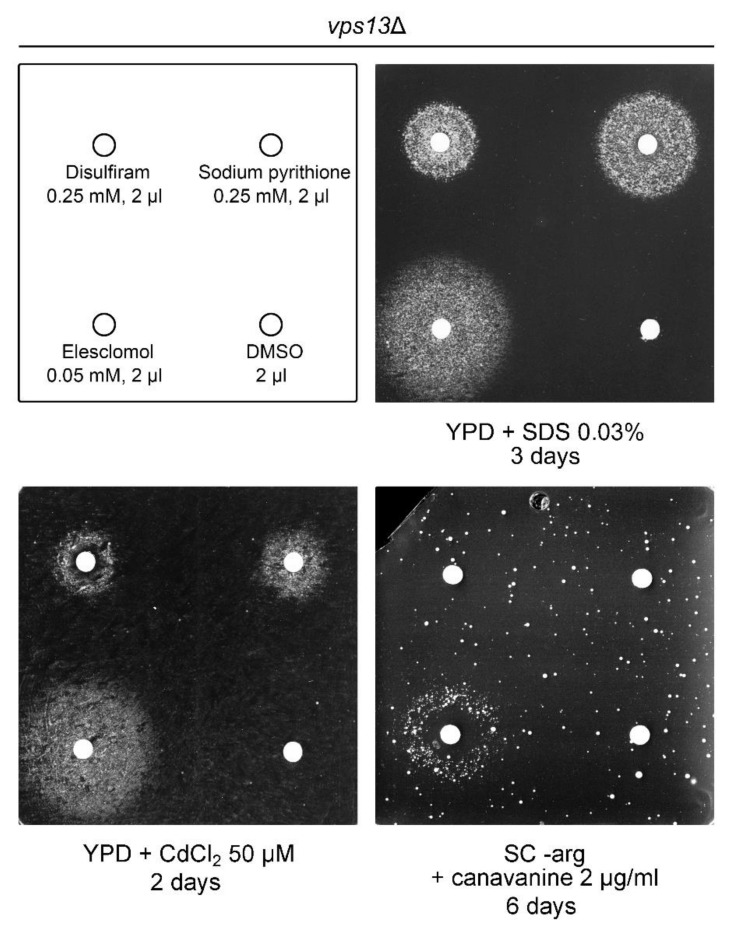
Copper ionophores suppress several phenotypes of *vps13*Δ. The *vps13*Δ mutant cells were spread onto the plates with respective media and tested substances were spotted on the filter papers. The order of the substances and concentrations used are indicated. Dimethyl sulfoxide (DMSO) was used as a negative control.

The copper ionophore which origins from the Prestwick library was disulfiram (Figure 5), a drug originally used in treatment of alcoholism and currently gaining interest as a potential anticancer drug with several clinical trials ongoing [64]. The anticancer activity of disulfiram depends on its ability to increase intracellular copper content in cancer cells [65,66,67,68,69]. Disulfiram improved cytochrome c oxidase activity in models of Menkes disease [70] and was also shown to rescue respiratory growth defects related to copper deficiency in yeast [71].

The next tested copper ionophore, which we selected after performing the screen, was elesclomol (Figure 5), an anticancer candidate drug [72,73,74,75,76,77,78] that increases intracellular copper content with high selectivity to mitochondria [79,80] and targets electron transport chain [81,82]. It was also shown to rescue defects observed in various copper deficiency models, including Menkes disease model [80,83].

The last selected copper ionophore was a sodium salt of pyrithione (Figure 5). A different pyrithione salt, zinc pyrithione, is an antimicrobial agent that inhibits yeast growth through copper influx [84]. It was proposed that the pyrithione salt dissociates, forms complex with extracellular copper and then enters cells [84]. We used sodium salt of pyrithione, which is less toxic to yeast [84]. Moreover, zinc chloride was highly toxic to yeast on YPD + SDS medium (our unpublished results); therefore, we wanted to avoid zinc salts in our experiments. Sodium pyrithione was also shown to improve mitochondrial ATP synthase activity in models of ATP synthase deficiencies by targeting mitochondrial protein import [85].

**Figure A2:**
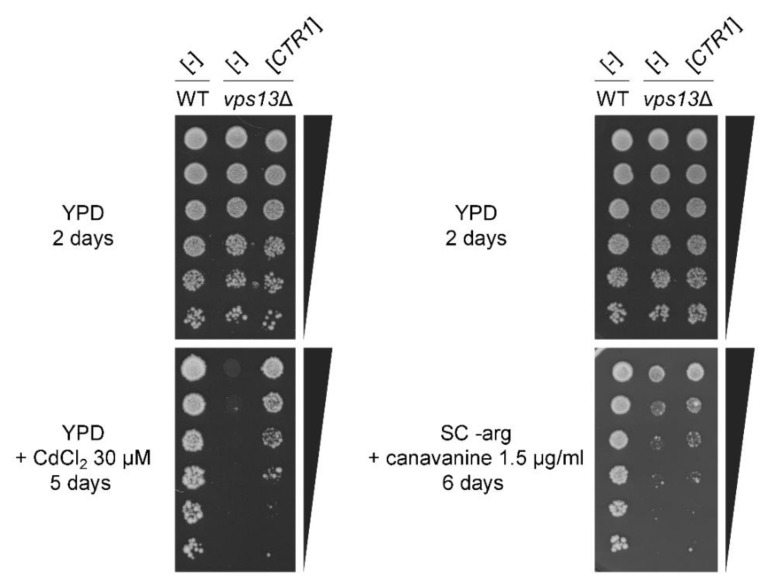
*CTR1* overexpression suppresses *vps13*Δ hypersensitivity to cadmium and very weakly suppresses hypersensitivity to canavanine. Growth of wild type and *vps13*Δ strains bearing plasmid with *CTR1* gene or empty vector was compared by a drop test on plates with YPD, YPD + CdCl_2_ and SC –arg + canavanine media.

All three copper ionophores improved growth of *vps13*Δ cells (Figure 5). However, the sizes of the growth zones were different for all the drugs, indicating different activities with elesclomol yielding the biggest growth zone at the lowest concentration. Moreover, suppression by the copper ionophores was not limited to SDS-hypersensitivity phenotype only. We tested two other growth phenotypes of *vps13*Δ, which we described previously: cadmium hypersensitivity [57] and canavanine hypersensitivity, implying a defect of endocytosis in *vps13*Δ [50]. All copper ionophores were active when tested for the reversing growth defect of *vps13*Δ cells on medium with cadmium (Figure 5) [57], whereas elesclomol was the only compound that improved *vps13*Δ growth on medium with canavanine (Figure 5) [50]. We also checked if *CTR1* overexpression suppresses *vps13*Δ hypersensitivity to cadmium and canavanine. The *vps13*Δ growth on media with cadmium addition was improved upon *CTR1* overexpression (Figure A2, left panel). For the canavanine hypersensitivity phenotype, *vps13*Δ suppression by *CTR1* was very weak (Figure A2, right panel). These results show that suppression of *vps13*Δ with copper ionophores or *CTR1* overexpression is not specific to SDS, but more general.

### 2.6. Copper Sulphate and Elesclomol Require FET3, but Not CCC2, for vps13Δ Suppression, While Disulfiram and Sodium Pyrithione Require Both FET3 and CCC2

**Figure 6:**
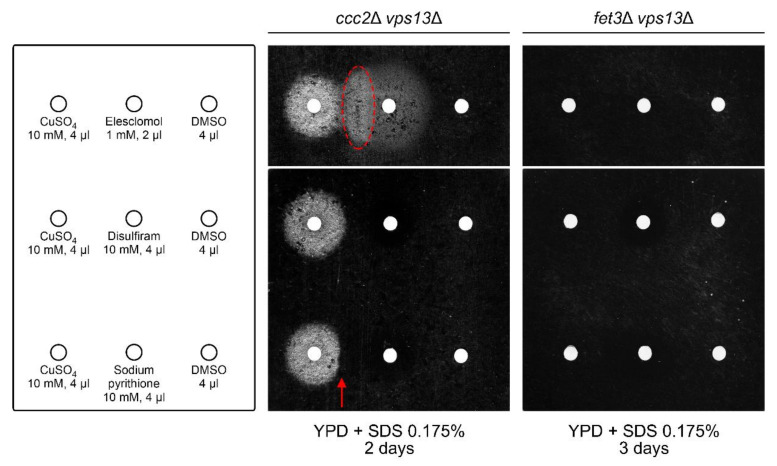
Impact of copper ionophores on the growth of *ccc2*Δ *vps13*Δ and *fet3*Δ *vps13*Δ cells on YPD + SDS media. Respective strains were spread onto the plates and substances were spotted on the filter papers. The order of the substances and concentrations used are indicated. DMSO was used as a negative control. The dashed ellipse in red indicates the enhanced zone of growth in the border between copper sulphate and elesclomol application. The red arrow indicates the zone of growth suppression in the border between copper sulphate and sodium pyrithione application.

To check whether the high-affinity iron uptake system is also required for suppression of *vps13*Δ by copper sulphate and copper ionophores, we tested their effects on growth of *ccc2*Δ *vps13*Δ and *fet3*Δ *vps13*Δ mutant strains on media with SDS. Surprisingly, copper sulphate and elesclomol improved growth of *ccc2*Δ *vps13*Δ strain (Figure 6), while disulfiram and sodium pyrithione were not active. Moreover, we observed a disc-shaped area of more prominent growth inside the growth zone caused by elesclomol (indicated on Figure 6). The fact that this area was on the side of copper sulphate application site indicates positive interaction of elesclomol and copper sulphate in suppression of *ccc2*Δ *vps13*Δ. To observe the activity of these compounds in the *ccc2*Δ *vps13*Δ mutant, higher concentrations were used than in previous experiments with *vps13*Δ strain (20× more than in Figure 1b and Figure 5). Disulfiram and sodium pyrithione were also tested in higher concentrations (80× more than in Figure 5), but no growth zones were observed (Figure 6). We noticed also a narrowed growth zone around the filter with copper sulphate at the side of sodium pyrithione application site (indicated on Figure 6). This suggests an interaction between copper and sodium pyrithione that negatively affects *ccc2*Δ *vps13*Δ growth. When copper ionophores and copper sulphate were tested on *fet3*Δ *vps13*Δ strain, none of them restored its growth on YPD + SDS medium (Figure 6). This result shows that the high-affinity iron uptake system is required for suppression of *vps13*Δ by copper sulphate and copper ionophores, likewise for *CTR1*-based suppression. Interestingly, copper sulphate and elesclomol overcome the lack of *CCC2*.

**Figure 7:**
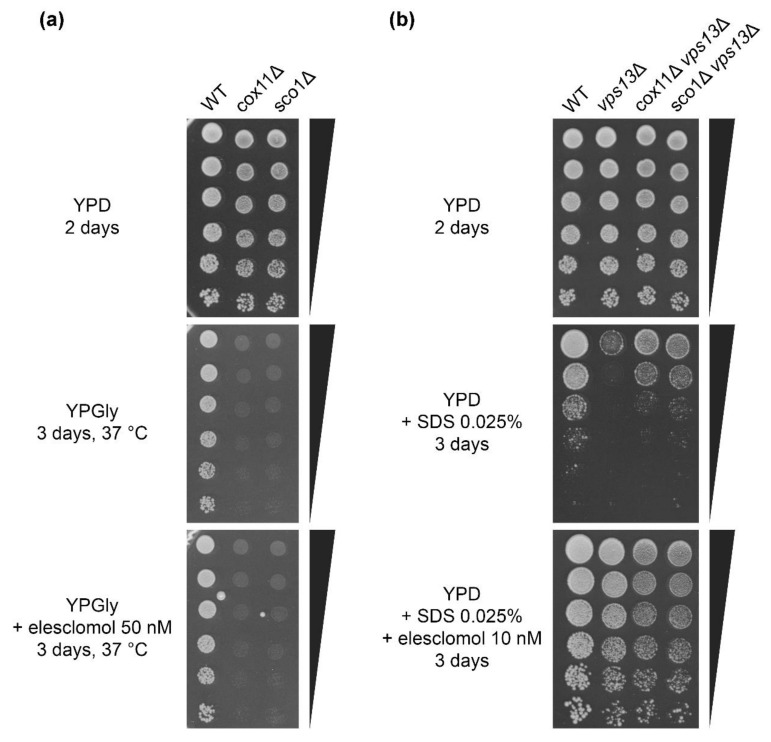
Elesclomol does not restore respiratory growth of yeast when CcO is inactivated, but CcO is not relevant for elesclomol suppression of *vps13*Δ on YPD + SDS media. (**a**) *cox11*Δ and *sco1*Δ respiratory growth defect is not restored by elesclomol. Growth of wild type, *cox11*Δ and *sco1*Δ strains was compared by a drop test on plates with YPD and glycerol-containing YPGly and YPGly + elesclomol media in 30 °C or 37 °C. (**b**) *COX11* and *SCO1* are not required for elesclomol effect on *vps13*Δ growth on YPD + SDS media. Growth of wild type, *vps13*Δ, *cox11*Δ *vps13*Δ, and *sco1*Δ *vps13*Δ strains was compared by a drop test on plates with YPD, YPD + SDS and YPD + SDS + elesclomol media.

As mentioned in the previous chapter, the action of copper ionophores in other diseases models is linked with improvement of mitochondria function. Elesclomol was shown to improve activity of cytochrome c oxidase (CcO) and rescue respiratory growth defect of yeast mutants defective in copper metabolism (*coa6*Δ, *sco2*Δ, *cox12*Δ, *ctr1*Δ, *atx1*Δ, *ccs1*Δ, *gsh1*Δ, *ccc2*Δ, *gef1*Δ), however, not *sco1*Δ [80]. Sco1 is a copper-binding protein of mitochondrial inner membrane that delivers copper to the Cox2 subunit of CcO [86,87] and is required for CcO assembly [88,89]. Therefore the lack of growth improvement of *sco1*Δ after elesclomol treatment shows that CcO is a target of elesclomol action [80]. We confirmed this result and showed that Cox11, another protein delivering copper to CcO and required for CcO activity [90,91,92,93], is also essential for restoring respiratory growth by elesclomol (Figure 7a). To check if CcO is also relevant for suppression of *vps13*Δ on YPD + SDS media, we tested the effect of elesclomol treatment on the growth of *cox11*Δ *vps13*Δ and *sco1*Δ *vps13*Δ mutants. Growth of both mutants was improved (Figure 7b), which shows that elesclomol does not require *COX11* and *SCO1* for suppression of *vps13*Δ. Moreover, *cox11*Δ *vps13*Δ and *sco1*Δ *vps13*Δ strains were less sensitive to SDS than *vps13*Δ (Figure 7b), indicating that switching off CcO actually improves *vps13*Δ growth. These results show that CcO activity is not relevant for *vps13*Δ suppression by elesclomol and is not required for growth on YPD + SDS media. This also highlights the importance of the high-affinity iron uptake system for suppression of *vps13*Δ by copper ionophores.

## 3. Discussion

**Figure 8:**
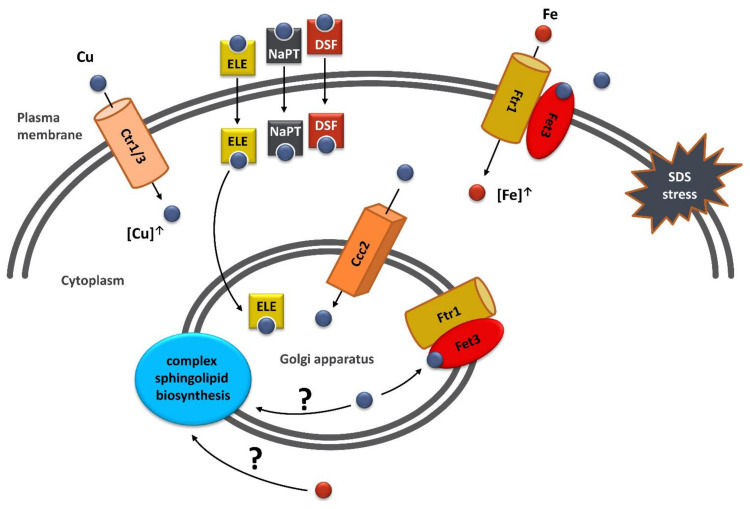
Schematic representation of the proposed model of copper-based suppression mechanism of *vps13*Δ SDS hypersensitivity phenotype. Stress triggered by SDS in the cytoplasm and in the plasma membrane is suppressed by increased copper uptake either by overexpression of the genes of the plasma membrane copper transporters Ctr1 or Ctr3, or by treatment with copper ionophores (ELE—elesclomol; NaPT—sodium pyrithione or DSF—disulfiram). Internalized copper is transported to the Golgi apparatus by Ccc2 ATPase or by elesclomol, which is also active even in the absence of Ccc2. Then, copper is incorporated into Fet3 oxidase, which, together with Ftr1 permease, forms a complex responsible for the high-affinity iron uptake. This complex is transported to the plasma membrane, where it facilitates iron uptake. Iron is essential for protection against SDS stress and iron deficiency impairs lipid synthesis, altering the properties and functions of cellular membranes. When copper is abundant in the environment, it could be also directly incorporated into Fet3 localised on the plasma membrane. Another possible (yet not investigated here) putative mechanism of copper action is its direct impact on complex sphingolipid biosynthesis in Golgi apparatus, which are important for SDS stress response [57].

Ion homeostasis is crucial for proper functioning of all organisms, from unicellular eukaryotes, such as *Saccharomyces cerevisiae* yeast, to humans, in which ion imbalance is associated with many diseases, including neurodegenerative ones. In order to improve patients’ health, one should understand the mechanisms of these diseases, which would enable the development of effective therapies. For that, model organisms are used and studies on them can provide valuable results due to the evolutionary conservation of cellular mechanisms responsible for ion homeostasis. Here, we showed that defect(s) observed in the yeast model of *VPS13*-related diseases can be overcome by overexpression of *CTR1* or *CCC2* genes or copper salts addition. We analysed potential mechanisms of copper-based suppression of *vps13*Δ, and we found that it acts by increasing iron level via the high-affinity iron uptake system. We investigated functioning of the high-affinity iron uptake system and found that the levels of its components, Ccc2 and Fet3, which are required for *CTR1*-based suppression, were elevated in *vps13*Δ. Finally, we presented three copper ionophores, that suppressed *vps13*Δ growth defect, and we showed that they also required the functional high-affinity iron uptake system (*FET3* and *CCC2* genes) for their action with the exception of elesclomol, which did not require *CCC2*. Our results point to the copper-dependent iron uptake as a suppression mechanism of *vps13*Δ defects and a potential drug target in *VPS13*-depenedent diseases (Figure 8). However, we cannot exclude that other mechanisms may also contribute in *vps13*Δ suppression.

Our findings on the *CTR1* and *CTR3* genes, encoding copper transporters, and copper sulphate as suppressors of the *vps13*Δ phenotype turned our attention to the functioning of copper homeostasis in these cells. In *vps13*Δ, Ctr1–GFP was located at the plasma membrane, as in wild type cells, but in addition, we observed strong vacuolar GFP signal. This corresponded well with the western blot analysis that showed similar full-length Ctr1–GFP levels but more degradation products, including free GFP in *vps13*Δ cell extracts as compared with the wild type. This increased degradation of Ctr1–GFP could be caused by protein trafficking defects observed in *vps13*Δ, such as defects in retromer functioning [43,45,50]. Retromer is possibly required in yeast Ctr1 recycling from endosomes to plasma membrane, as it was recently documented for Ctr1 in mammalian cells [94]. Decreased recycling can cause increased transport of Ctr1–GFP from endosomes to the vacuole. The other possibility is that degradation of Ctr1–GFP is increased in *vps13*Δ cells due to the elevated copper content. It was shown that Ctr1 is degraded when copper is abundant [28,29]; therefore, this explanation could also be possible. However, the simplest mechanism of copper-based suppression of *vps13*Δ would be by compensating the putative decrease in copper level. To clarify this contradiction, copper content in wild type and mutant cells was studied, and we did not find indication of copper deficiency in the *vps13*Δ mutant in tested conditions. Moreover, a slight increase in copper content was observed, which may indicate increased demand for copper and effective copper transport in *vps13*Δ cells. This also supports the possibility of increased Ctr1–GFP degradation due to the elevated copper level. One should also keep in mind that copper levels were determined in yeast grown in YPD media supplemented with copper salt, therefore copper entered the cell via other transporters than Ctr1 [23]. To conclude this part, we observed increased degradation of Ctr1–GFP. However, since copper level in *vps13*Δ is not lowered in tested conditions, mechanism of *CTR1* suppression is rather different than compensating putative copper deficiency.

Since we did not detect any copper deficiency that could be compensated by increasing copper transport, another mechanism of copper-related suppression of *vps13*Δ phenotype must exist. Based on our previous results regarding the importance of iron for SDS tolerance in yeast cells [57], we hypothesised that excessive copper can be utilised in Fet3 multicopper oxidase in order to increase iron uptake and, therefore, improve *vps13*Δ growth. We confirmed this by showing that *CTR1* overexpression caused an increase in iron levels in *vps13*Δ cells and by the fact that *FET3* and *CCC2* genes were essential for *vps13*Δ suppression by *CTR1*. We also showed that overexpression of *CCC2* suppresses the growth defect of *vps13*Δ, but not *fet3*Δ *vps13*Δ. This suggests that increased iron level in *vps13*Δ can be also achieved by increasing import of copper to the Golgi apparatus; however, our iron measurement experiments did not provide solid proof. An increase in iron content in *vps13*Δ upon *CCC2* overexpression was relatively low (17%, compared to 66% for *CTR1*) and did not cross the statistical significance threshold (*p* = 0.066). The hypothesis that *CCC2* can also act by increasing iron content via the high-affinity iron uptake system was supported by the fact that *FET3* is required for *CCC2*-based suppression. Moreover, results of other studies show that deletion of *CCC2* causes defective iron uptake [13,95]. Finally, *CCC2* overexpression rescues growth defect in iron-limited conditions of *atx1*Δ mutant [95], which is devoid of cytoplasmic Atx1 copper chaperone involved in copper delivery to Ccc2 [95]. However, other potential non-iron-related mechanisms of *CCC2* action may exist. Addition of copper sulphate greatly increased iron content in cells, showing that copper alone may act via the same mechanism as copper transporters. When we tested the impact of *CTR1* and *CCC2* overexpression on iron levels in *vps13*Δ cells cultivated in a copper-rich environment, significant yet moderate increases in iron content (15% and 24% for *CTR1* and *CCC2*, respectively) were observed. Limited effects of *CTR1* and *CCC2* overexpression could be explained by achieving high copper saturation of Fet3 oxidase and reaching activity close to its limit in *vps13*Δ cells due to the copper supplementation. Therefore, further increases in intracellular copper by *CTR1* overexpression or improving copper import to the Golgi apparatus by *CCC2* overexpression may not greatly influence Fet3 activity and iron uptake in a copper-rich environment. To sum up, we propose that copper sulphate addition and overexpression of the *CTR1* and likely *CCC2* genes suppress *vps13*Δ by increasing iron content in cells. However, it was reported that deletion of *CCC2* or *FET3* causes copper sensitivity [96,97]; therefore, other putative, non-iron-related, beneficial effects of increased intracellular copper in *vps13*Δ cells may be impossible to observe in *ccc2*Δ *vps13*Δ and *fet3*Δ *vps13*Δ strains due to the fact of their sensitivity to copper.

Our analysis showed a lower level of iron in the *vps13*Δ mutant when compared with wild type cells. This is in agreement with the previous report, in which iron uptake was decreased in many *vps* mutants including *vps13* [14]. Copper supplementation increased iron content in *vps13*Δ cells; however, it was not as effective as it was in the wild type. When cultivating in YPD media, the iron level in *vps13*Δ was 74% of the wild type, while in YPD + CuSO_4_ it was only 45%. This may suggest partial insufficiency of the high-affinity iron uptake system in *vps13*Δ. Therefore, we investigated localisation and levels of protein components of this system: Ccc2 and Fet3. The localisation of Ccc2–GFP and Fet3–GFP proteins was not altered, but their levels were elevated, which may suggest greater demand for iron in *vps13*Δ. However, in our previous studies we showed that *vps13*Δ was not hypersensitive to ferrozine, an iron chelator limiting iron availability for cells, and signalling pathways responding to iron deficiencies were not activated in *vps13*Δ [57]. Moreover, *ccc2*Δ *vps13*Δ and *fet3*Δ *vps13*Δ double mutants were more sensitive to SDS than *vps13*Δ, which further implies that the high-affinity iron uptake system is functional in *vps13*Δ. Perhaps, elevated levels of Ccc2–GFP and Fet3–GFP proteins counteract lower iron content observed in *vps13*Δ. This decrease in iron level, however, is nowhere near the iron deficiency observed in the *fet3*Δ mutant. The *fet3*Δ cells are hypersensitive to SDS, but *vps13*Δ cells are much more so [57]. Therefore, lower iron level may contribute to *vps13*Δ poor growth on YPD + SDS media only to a small extent, next to previously described factors potentially related to SDS-hypersensitivity, such as impaired calcium homeostasis, altered cell wall integrity pathway or sphingolipid biosynthesis [51,57]. To conclude, our findings did not point to defective functioning of the high-affinity iron uptake system in *vps13*Δ; however, they suggest its partial insufficiency when copper is abundant.

Surprisingly, contrary to suppression by *CTR1* and *CCC2*, copper sulphate alone was active in *ccc2*Δ *vps13*Δ when a higher concentration was used. In other studies regarding copper, iron, Ccc2 and Fet3 interplay, it was shown that *ccc2*Δ is deficient in Fet3 oxidase activity, which results in impaired iron uptake and respiratory growth [13] and all these defects of *ccc2*Δ are reversed by supplementing growth media with copper [13,14,30,63]. It is possible that in *ccc2*Δ strain Fet3 acquires copper when is in the plasma membrane from copper-rich environment. This is further supported by the fact, that copper supplementation restores iron uptake in *ccc2*Δ in Ctr1-independent manner [14]. Therefore, incorporating environmental copper into Fet3 oxidase localised in the plasma membrane could be the mechanism in which copper suppress *ccc2*Δ *vps13*Δ SDS-hypersensitivity phenotype. No growth improvement of *fet3*Δ *vps13*Δ by copper was observed, which is in line with cited studies [13,30,63] and with the fact that no iron increase was observed in the *fet3*Δ strain upon copper supplementation.

Suppression of the *vps13*Δ was also achieved by treatment with copper ionophores and their action required the functional high-affinity iron uptake system. Interestingly, elesclomol, contrary to disulfiram and sodium pyrithione, was active also in *ccc2*Δ *vps13*Δ strain. Observed positive interaction of elesclomol and copper sulphate indicates that elesclomol activity in *ccc2*Δ *vps13*Δ is copper dependent. In other studies, elesclomol was shown to improve respiratory growth defect of *ccc2*Δ mutant [80]. Moreover, elesclomol alleviates defects observed in murine model of Menkes disease [83] in which function of ATP7A, a homologue of yeast Ccc2, is impaired. These data together with our results suggest that elesclomol may replace Ccc2 in its function, and transfer copper to the Golgi apparatus in *vps13*Δ. We also observed that elesclomol was the only drug among copper ionophores tested, which suppressed *vps13*Δ canavanine hypersensitivity phenotype, suggesting that this drug may improve endocytosis in *vps13*Δ or prevent canavanine-related toxic effects observed in yeast, such as proteotoxic stress [98]. We hypothesised that this is caused by elesclomol-bound copper ability to enter the Golgi apparatus; however, overexpression of *CCC2* did not improve *vps13*Δ growth on media with canavanine (not shown) indicating that elesclomol may reverse *vps13*Δ canavanine hypersensitivity growth defect in the Golgi-independent manner. We also tested the possibility that CcO could be a target of action of elesclomol, which activities are often linked with mitochondria functioning. We showed that the *COX11* and *SCO1* genes, crucial for CcO activity, were not required for elesclomol suppression of the *vps13*Δ SDS-hypersensitivity phenotype. Moreover, deletion of these genes improved growth of *vps13*Δ. We hypothesise that switching off CcO activity, a copper demanding process, may increase a cytoplasmic coper pool, therefore contributing to growth improvement. Summarising this part, our results show that copper ionophores suppress *vps13*Δ and improve its growth on YPD + SDS via the high-affinity iron uptake system.

While in this work we focused on copper-dependent iron uptake as an *vps13*Δ suppression mechanism, other copper dependent-mechanisms may exist. An interesting area for further investigation is the role of Ccc2 in sphingolipid biosynthesis. It was shown that cells lacking *CCC2* are defective in hydroxylation of complex sphingolipids [99]. Since elesclomol delivers copper to the Golgi apparatus [83], it may also influence synthesis of complex sphingolipids. It is still, however, unknown which enzyme is directly responsible for sphingolipids hydroxylation [100]. Noteworthy, the Sur2 protein required for hydroxylation of ceramides is a diiron enzyme [101], therefore increasing iron content could also influence complex sphingolipids synthesis. Moreover, *CCC2* was originally found as a cross-complementing gene of calcium sensitivity defect of *csg1* mutant [12]. *CSG1* encodes a catalytic subunit of the mannosylinositol phosphorylceramide synthase—an enzyme important for synthesis of sphingolipids. This further supports Ccc2 impact on sphingolipid biosynthesis and, perhaps, in some way on calcium signalling. Moreover, in previous studies, mutants defective in sphingolipid biosynthesis were shown to be hypersensitive to SDS [57,102]. These results imply that sphingolipid biosynthesis may be a potential target in improving *vps13*Δ functioning by copper-dependent mechanism directly or indirectly by affecting iron level.

To conclude, in this study, we showed that suppression of the *vps13*Δ SDS-hypersensitivity phenotype could be achieved by increasing the intracellular copper content, both in a genetic and pharmacological manner. We showed the importance of the high-affinity iron uptake system for the mechanism of copper-based suppression. While the iron system remains functional in *vps13*Δ cells, we did show some alteration in the levels of proteins involved in this system. This points to iron homeostasis as an interesting area for further investigation in higher model organisms of *VPS13*-related diseases. We also presented three copper ionophores as drugs effective in our *vps13*Δ yeast disease model. The fact that they are known substances, used in treatment for other diseases or are candidate drugs in ongoing clinical trials, make them particularly interesting for further testing. If their effectiveness is confirmed in higher model organisms, repurposing of these drugs will significantly ease their usage in patients, which is crucial especially for treatment of rare diseases.

## 4. Materials and Methods

### 4.1. Strains, Media, and Growth Conditions

The *Escherichia coli* DH5α strain was used for plasmid propagation. The *Saccharomyces cerevisiae* strains used are listed in Table 1. YPD complete medium (1% yeast extract (Biokar Diagnostics, Allonne, France), 2% tryptone (Biokar Diagnostics), 2% glucose, 1% adenine), glycerol-containing YPGly (2% glycerol) medium or synthetic complete (SC) medium (0.067% yeast nitrogen base without amino acids, 2% glucose) with desired supplementation (amino acids, uracil, adenine, salts, and drugs) were used for yeast cultivation. For drop tests, cells were grown overnight in respective liquid media, and cultures were diluted in sterile distilled water to obtain a cell optical density (OD) equal to OD600∼1. Subsequently, aliquots of 4-fold serial dilutions of cultures were spotted onto solid media. Plates were incubated at 30 °C or 37 °C if indicated. Like in our previous studies [51,57], growth experiments on YPD + SDS plates were performed using several concentrations of SDS and images were taken after different time of incubation to show representative results. This approach was necessary because of the high sensitivity of yeast cells to small changes in the final SDS concentration in the plate, which depends on the lot of medium and SDS stock as well as other physical factors.

*The ccc2*Δ *vps13*Δ, *fet3*Δ *vps13*Δ, *cox11*Δ *vps13*Δ and *sco1*Δ *vps13*Δ strains were constructed using a *vps13*::*URA3* cassette. The cassette was amplified by PCR using pKA475 plasmid (Table 2) as a template. Respective strains were transformed with the cassette and plated on selective media. Integration was confirmed by PCR.

Strains producing C-terminally GFP-tagged proteins (Ctr1–GFP, Ccc2–GFP, Fet3–GFP) were constructed according to a previously describe method [103]. The cassettes were amplified by PCR using pFA6a-GFP(S65T)-kanMX6 (Table 2) as a template. BY4741 and KJK181A (*vps13::URA3*) strains were transformed with obtained cassettes and were plated on selective media. Integrations were confirmed by PCR.

### 4.2. Plasmids

The plasmid used are listed in Table 2. pFL44-CTR3, containing the *CTR3* gene and fragments of the *VIP1* and *BER1* genes was isolated from the pFL-44 genomic bank [61] in our screen for multicopy suppressors of *vps13-I2749R* [51]. pYEp181lac-CTR3 was obtained by amplifying the *CTR3* gene by PCR using pFL44-CTR3 as a template and ligation of the PCR product to pYEp181lac digested with *EcoR*I and *BamH*I restriction enzymes. pYEp181lac-CTR1 and pYEp181lac-CCC2 were obtained by amplifying the *CTR1* and *CCC2* genes by PCR using genomic DNA as a template and subsequent ligation of the PCR products to YEp181lac digested with *Hin*dIII and *Pst*I restriction enzymes for *CTR1* and *Xba*I, and *EcoR*I for *CCC2*.

pRS415-SEC7-mRFP was obtained by transferring fragment from pRS316-SEC7-mRFP to pRS415 using *BamH*I and *Xho*I restriction enzymes.

### 4.3. Copper and Iron Measurements

Our protocol was adapted based on previously used approaches [108]. Briefly, the BY4741 and *vps13*Δ (KJK181A) yeast strains, bearing empty vector or plasmid expressing the *CTR1* or *CCC2* genes, were grown for 24 h in YPD + 0.25 mM CuSO_4_ for copper measurement and in YPD or YPD + 0.25 mM CuSO_4_ for iron measurement. Copper supplementation was required to observe results above copper levels in blank samples. After cultivation, cells were collected and washed once with 10 mM EDTA (ethylenediaminetetraacetic acid) and three times with metal-free water. Next, 16 OD of yeast cells (measured after washing) was digested in 95 °C for 24 h in 10% ultrapure nitric acid (HNO_3_) with occasional mixing. Digestion products were diluted 4 times with metal-free water and subsequently measured by inductively coupled plasma atomic emission spectroscopy (ICP-AES). Measurements were performed commercially in Laboratório de Análises at Universidade Nova de Lisboa (Department of Chemistry, Faculdade de Ciências e Tecnologia, Universidade Nova de Lisboa, 2829-516 Caparica, Portugal). Data were processed and visualized in Python (3.6.4, https://www.python.org/, accessed on 14 October 2020) using Pandas (1.0.1, The pandas development team, https://zenodo.org/record/3644238#.YDKvquhKg2w, accessed on 14 October 2020) and Seaborn (0.11.0, Michael Waskom and the seaborn development team, https://zenodo.org/record/4019146#.YDKwNuhKg2x, accessed on 14 October 2020) libraries. Statistical analysis was performed with R (Rcmdr, 2.6–2, https://www.rcommander.com/, accessed on 14 October 2020).

### 4.4. Drug Tests

Drug tests were performed as described previously [57,109], with the exception for the canavanine hypersensitivity phenotype, where yeast were cultivated in SC –arg media to exponential phase prior spreading on SC –arg + canavanine plate. Briefly, cells were spread onto plates with the desired solid media, sterile paper filters were placed on the surface of the media, and drugs were applied on filters. Originally, disulfiram was identified as an active drug from Prestwick Chemical Library (Prestwick Chemical, Illkirch-Graffenstaden, France; last used 04.2017). In the presented work, drugs were used from the undermentioned sources: disulfiram, sodium pyrithione, bathocuproinedisulfonic acid disodium salt, and ferrozine (Sigma–Aldrich, St. Louis, MO, USA), and elesclomol (MedChemExpress, Monmouth Junction, NJ, USA).

### 4.5. Fluorescence Microscopy

For fluorescence microscopy of cells producing Ctr1–GFP protein, yeast were grown in YPD for 24 h and were stained with CMAC (7-amino-4-chloromethylcoumarin) for 30 min prior to observation. For fluorescence microscopy of cells producing Ccc2–GFP and Fet3–GFP proteins, yeast were grown in SC –leu + 0.25 mM ferrozine (an iron chelator limiting iron availability for cells) to early exponential phase (5 h of cultivating). Strains expressing genomic copy of *CCC2–GFP* were transformed with plasmid encoding Sec7–RFP. For the Fet3–GFP localization studies, strains with GFP-tagged Fet3 were transformed with empty vector. Cells were viewed with an Eclipse E800 (Nikon, Tokyo, Japan) fluorescence microscope equipped with a DS-5Mc camera (Nikon, Tokyo, Japan). Images were collected using Lucia General 5.1 software (Laboratory Imaging Ltd., Prague, Czech Republic). The same fields were viewed by differential interference contrast (DIC) optics.

### 4.6. Western Blotting

For western blotting, yeast were grown in the same conditions as for fluorescence microscopy: 24 h in YPD for strains with Ctr1–GFP and 5 h in SC –leu for strains (bearing empty vector) with Ccc2–GFP and Fet3–GFP. After cultivation, cells were disrupted with glass beads (0.5 mm) in 2x Thorner buffer and incubated in 37 °C for 10 min. Samples were analysed by SDS-PAGE followed by western blotting using anti-GFP (1:4000, Roche, Basel, Switzerland), anti-Pgk1 (1:20,000, Thermo Fisher Scientific, Waltham, MA, USA) and anti-mouse horseradish peroxidase (HRP)-conjugated (1:4000, Jackson ImmunoResearch, Baltimore Pike, PA, USA) antibodies. Blots were developed using Pierce™ enhanced chemiluminescence (ECL) Western Blotting Substrate (Thermo Fisher Scientific, Waltham, MA, USA), except for Ccc2–GFP protein, where Amersham™ ECL™ Prime Western Blotting Detection Reagent (GE Healthcare, Chicago, IL, USA) was used. Densitometry was performed with ImageJ (https://imagej.nih.gov/ij/, accessed on 14 October 2020). Data were processed and visualized in Python (3.6.4, https://www.python.org/, accessed on 14 October 2020) using Pandas (1.0.1, The pandas development team, https://zenodo.org/record/3644238#.YDKvquhKg2w, accessed on 14 October 2020) and Seaborn (0.11.0, Michael Waskom and the seaborn development team, https://zenodo.org/record/4019146#.YDKwNuhKg2x, accessed on 14 October 2020) libraries. Statistical analysis was performed with R (Rcmdr, 2.6–2, https://www.rcommander.com/, accessed on 14 October 2020).

## Figures and Tables

**Table 1:** Strains used in this study.

Strain	Genotype	Source
BY4741	MAT**a** *his3*Δ*1 leu2*Δ*0 met15*Δ*0 ura3*Δ*0*	Open Biosystem
BYvps13Δ	MAT**a** *his3*Δ*1 leu2*Δ*0 met15*Δ*0 ura3*Δ*0 vps13*::*kanMX*	Open Biosystem
KJK181A	MAT**a** *his3*Δ*1 leu2*Δ*0 met15*Δ*0 ura3*Δ*0 vps13::URA3*	[57]
BYctr1Δ	MAT**a** *his3*Δ*1 leu2*Δ*0 met15*Δ*0 ura3*Δ*0 ctr1*::*kanMX*	Open Biosystem
BYccc2Δ	MAT**a** *his3*Δ*1 leu2*Δ*0 met15*Δ*0 ura3*Δ*0 ccc2*::*kanMX*	Open Biosystem
PS13	MAT**a** *his3*Δ*1 leu2*Δ*0 met15*Δ*0 ura3*Δ*0 ccc2*::*kanMX vps13::URA3*	This study
BYfet3Δ	MAT**a** *his3*Δ*1 leu2*Δ*0 met15*Δ*0 ura3*Δ*0 fet3*::*kanMX*	Open Biosystem
PS14	MAT**a** *his3*Δ*1 leu2*Δ*0 met15*Δ*0 ura3*Δ*0 fet3*::*kanMX vps13::URA3*	This study
BYcox11Δ	MAT**a** *his3*Δ*1 leu2*Δ*0 met15*Δ*0 ura3*Δ*0 cox11*::*kanMX*	Open Biosystem
PS5	MAT**a** *his3*Δ*1 leu2*Δ*0 met15*Δ*0 ura3*Δ*0 cox11*::*kanMX vps13::URA3*	This study
BYsco1Δ	MAT**a** *his3*Δ*1 leu2*Δ*0 met15*Δ*0 ura3*Δ*0 sco1*::*kanMX*	Open Biosystem
PS6	MAT**a** *his3*Δ*1 leu2*Δ*0 met15*Δ*0 ura3*Δ*0 sco1*::*kanMX vps13::URA3*	This study
PS7	MAT**a** *his3*Δ*1 leu2*Δ*0 met15*Δ*0 ura3*Δ*0 CTR1-GFP*::*kanMX*	This study
PS8	MAT**a** *his3*Δ*1 leu2*Δ*0 met15*Δ*0 ura3*Δ*0 CTR1-GFP*::*kanMX vps13::URA3*	This study
PS9	MAT**a** *his3*Δ*1 leu2*Δ*0 met15*Δ*0 ura3*Δ*0 CCC2-GFP*::*kanMX*	This study
PS10	MAT**a** *his3*Δ*1 leu2*Δ*0 met15*Δ*0 ura3*Δ*0 CCC2-GFP*::*kanMX vps13::URA3*	This study
PS11	MAT**a** *his3*Δ*1 leu2*Δ*0 met15*Δ*0 ura3*Δ*0 FET3-GFP*::*kanMX*	This study
PS12	MAT**a** *his3*Δ*1 leu2*Δ*0 met15*Δ*0 ura3*Δ*0 FET3-GFP*::*kanMX vps13::URA3*	This study

**Table 2:** Plasmids used in this study.

Plasmid	Source
pYEp181lac	[104]
pFL44-CTR3 (from FL44-based genomic bank)	This study
pYEp181lac-CTR3	This study
pYEp181lac-CTR1	This study
pYEp181lac-CCC2	This study
pFA6a-GFP(S65T)-kanMX6	[105]
pRS415	[106]
pRS316-SEC7-mRFP	[107], restricted
pRS415-SEC7-mRFP	This study, restricted
pKA475 (*vps13*Δ::*URA3*)	[57]

## Data Availability

The data that support the findings of this study are available from the corresponding authors upon reasonable request.
